# High Pressure Treatment in Foods

**DOI:** 10.3390/foods3030476

**Published:** 2014-08-19

**Authors:** Edwin Fabian Torres Bello, Gerardo González Martínez, Bernadette F. Klotz Ceberio, Dolores Rodrigo, Antonio Martínez López

**Affiliations:** 1 Institute of Agrochemistry and Food Technology (CSIC), Avenida Agustín Escardino, 7 Parque Científico, 46980 Paterna (Valencia), Spain; E-Mails: lolesra@iata.csic.es (D.R.); amartinez@iata.csic.es (A.M.L.); 2 Alpina Research Institute (IAI), Alpina Productos Alimenticios S.A, Edificio Corporativo Km 3 vía, Briceño-Sopó, Cundinamarca, 251001, Colombia; E-Mails: gerardo.gonzalez@alpina.com.co (G.G.M.); bernadette.klotz@alpina.com (B.F.K.C.)

**Keywords:** high pressure, microorganism, spores, protein, enzyme, packaging, cheese

## Abstract

High hydrostatic pressure (HHP), a non-thermal technology, which typically uses water as a pressure transfer medium, is characterized by a minimal impact on food characteristics (sensory, nutritional, and functional). Today, this technology, present in many food companies, can effectively inactivate bacterial cells and many enzymes. All this makes HHP very attractive, with very good acceptance by consumers, who value the organoleptic characteristics of products processed by this non-thermal food preservation technology because they associate these products with fresh-like. On the other hand, this technology reduces the need for non-natural synthetic additives of low consumer acceptance.

## 1. Introduction

Currently consumers worldwide are more demanding with regard to the quality and safety of the foods they consume, especially those that produce the perception of healthy products. To meet these demands, the food industry has improved its heat preservation processes by developing continuous high temperature/short time (HTST) and ultra high temperature (UHT) treatments and aseptic packaging. In addition, consumption of minimally processed products has increased significantly. These products maintain a high standard of nutrition and flavor, while meeting the required safety level and achieving a long shelf life [1].

Minimally processed foods have been developed alongside the development of various emerging preservation technologies. Within this group of technologies there are the so-called “non-thermal preservation technologies,” which do not use heat as the main form of microbial and enzyme inactivation. Although heat is generated by some of these processes, the temperature increase never reaches the levels of a conventional thermal process and can be suitably controlled by a cooling station. These new preservation technologies include oscillatory magnetic fields, pulsed electric fields, ultrasound, irradiation, and high hydrostatic pressure. Probably the most developed and most widely implanted technology at the industrial level is high hydrostatic pressure. This technology has demonstrated its capability of preserving sensory and nutritional qualities of foods while producing suitable levels of microbiological and enzyme inactivation.

## 2. High Hydrostatic Pressure Technology

The main objective of any non-thermal technology is to maximize the freshness and flavor qualities of the foodstuffs while achieving the required level of food safety. High hydrostatic pressure (HHP) meets with these requirements and today it being incorporated in many companies as an alternative to conventional heat treatment procedures. Applications include the preservation of meat products, oysters, fruit jams, fruit juices, salad dressings, fresh calamari, rice cake, duck liver, jam, guacamole, and many ready-to-eat foods. In all these cases, microbial and enzyme inactivation is achieved without altering the product quality [2]. In relation to the total percentage utilization of HHP equipment, vegetable products account for 28%, meat products for 26%, sea foods and fish for 15%, juices and beverages for 14%, and other products for 17%, generating an amount of 350,000,000 kg of processed products in 2012, according to data from Hiperbaric, S.A. [3].

All this makes HHP the most commercially developed non-thermal technology, with very good acceptance by consumers, who value the organoleptic characteristics of pressure-treated products with a quality barely affected by treatment. Currently the world market has experienced significant growth in the incorporation of equipment at industrial level (Table 1).

**Table 1:** Number of HHP machines around the world. Source: Hiperbaric, S.A. [3].

Time (Years)	HPP machines in industry
1990	2
1991	2
1992	3
1993	3
1994	4
1995	4
1996	4
1997	4
1998	5
1999	9
2000	14
2001	21
2002	27
2003	38
2004	52
2005	68
2006	78
2007	95
2008	109
2009	122
2010	147
2011	167

In general, microbial inactivation is achieved at pressures that vary from 100 to 800 MPa during relatively short times (from a few seconds to several minutes). Some treatments are combined with mild temperatures between 20 and 50 °C to inactivate enzymes. The processing conditions depend fundamentally on the food to be treated and the microorganisms and enzymes to be inactivated; we note that this technology at the pressure currently used in the food industry does not inactivate bacterial spores [4,5].

## 3. Packaging

The package is an important part in the development and industrial application of HHP as a preservation technology. It is possible to use a great variety of packages with different shapes; however, food must be packed in a flexible and resistant package, able to withstand pressure and maintain the integrity. Polyethylene (PE), polyethylene terephthalate (PET), polypropylene (PP), ethylene-vinyl alcohol (EVOH), polyamide (PA), and nylon films are some of the packaging materials currently used in industrial food processing by HHP treatments [6,7]. Juliano *et al.* [7] suggest minimize the headspace up to 30% to maximize the utilization of the vessel capacity and minimize the time needed for preheating, if the treatment requires temperature. Usually, an HPP vessel will utilize its 50%–70% volume capacity depending on the shape of the package and the vessel design [8].

## 4. Microbial Inactivation

The objective of any preservation process is the inactivation of microorganisms that can spoil the food and/or produce illness in the consumer (pathogenic microorganisms). The response of microorganisms to HHP has been extensively studied [9,10,11,12] varies according to the following factors: molds and yeasts are the most sensitive microorganisms; Gram-negative bacteria have medium sensitivity, whereas Gram-positive bacteria are the most resistant among vegetative cells and their spores need very high pressures to be inactivated. Regarding the action mechanisms of pressure, according to the studies carried out by Huang *et al.* [13] a pressure of 50 MPa can affect or inhibit protein synthesis and produce a reduction in the number of microbial ribosomes. A pressure of 100 MPa can cause partial denaturalization of cellular proteins; when the pressure is increased to 200 MPa it produces internal damage in the microbial structure and external damage in the cellular membrane. Pressures equal or similar to 300 MPa produce irreversible damage to the microorganism, including leakage of intracellular components to the surrounding medium, resulting finally in cellular death [14,15,16].

The various effects that take place in microorganisms depend on their physiological state, microorganisms in log phase being more sensitive to HHP than those in stationary phase. This behavior could be explained by the fact that in the log phase the microorganism is in the process of cellular division and the membrane is more sensitive to environmental stresses [6]. This effect was also reported by Mañas and Mackay [17] in *Escherichia coli* strain J1, in exponential and stationary phases. The cells in stationary phase showed higher resistance to HHP treatment than those in exponential phase. Some modifications were also observed (aggregation of cytoplasmic proteins, condensation of the nucleoid) after 200 MPa treatments for 8 min at 20 °C.

Temperature is a very important environmental stress in HHP treatments because the combination of the two technologies, with short times can increases significantly microbial inactivation. According to studies carried out by Chen and Hoover [18] and Ross *et al.* [19], an HHP treatment of* L. monocytogenes* at initial temperatures of 45–50 °C and 5 min produced more than 5 log decimal reductions in the initial microbial concentration in UHT whole milk. However, was necessary to increase the treatment time to 35 min to produce the same inactivation at initial temperature of 22 °C.

HHP has proved to be an effective technology for inactivating various pathogens, as reported by Jofré *et al.* [20]. The application of a treatment of 600 MPa for 6 min at 31 °C resulted in a reduction close to 3.5 decimal log for *E. coli*, *Listeria monocytogenes*, *Salmonella enterica* subsp. *enterica*, *Yersinia enterocolitica*, and *Campylobacter jejuni* in meat products.

Although there are many studies in relation to the effect of HHP on bacteria, the information that exists on molds and yeasts is relatively scarce (Table 2). In general, yeasts and molds can be inactivated at 200–400 MPa [21], but when they are in the spore or ascospore state or in a food with a very high concentration of sugar the pressure needed to inactivate them could be close to 600 MPa [22]. These microorganisms are frequently involved in spoilage of cereals derivatives (tofu, tortillas), minimally processed vegetables, and lactic derivatives such as butter, yoghurt, and soft cheese [23,24].

**Table 2:** High hydrostatic pressure (HHP) inactivation of molds and yeasts in different foods.

Food product	Microorganism	HHP conditions	Inactivation results	Reference
Pineapple juice	*Byssochlamys nivea*	550–600 MPa for 3–15 min at 20–80 °C	600 MPa for 15 min at 80 °C, 5.7 log reduction	Ferreira *et al.* [25]
Apple-broccoli juice	*S. cerevisiae*; *A. flavus*	250–400 MPa for 5–20 min at 21 °C	400 MPa for 10 min at 21 °C, 5 log reduction	Houška *et al.* [26]
Apple juice	*Talaromyces avellaneus*	200–600 MPa for 10–60 min at 17–60 °C	600 MPa for 50 min at 60 °C, 5 log reduction ascospores	Voldřich *et al.* [27]
Concentrated orange juice	*S. cerevisiae*	100–400 MPa for 0–120 min at 20 °C	400 MPa for 60 min at 20 °C, 3 log reduction	Basak *et al.* [28]
Cheese	*P. roqueforti*	50–800 MPa for 20 min at 10–30 °C	400 MPa for 20 min at 20 °C, 6 log reduction	O’Reilly *et al.* [29]

## 5. Spore Inactivation

Spores are cellular forms that some microorganisms have developed as a response to adverse environmental situations in order to survive. Spores are characterized by their high resistance to different environmental stresses and preservation treatments. The most important spore-producing genera are *Clostridium*, *Bacillus* and *Alicyclobacillus*. The initial spore load present in foods can be significantly reduced by HHP in combination with mild temperatures (Table 3). In various published studies, 3.5 decimal log reductions have been reported for *Clostridium sporogenes* and 5.7 decimal log reductions for *Bacillus coagulans* by HHP at temperatures of 60–90 °C [30,31]. Furthermore, Meyer [32] observed significant reductions in the initial spore concentration in low-acid foods after treatments ranging between 700 and 1000 MPa and a product temperature of 70 °C. With those conditions they obtained foods that were microbiologically stable at room temperature, and in many cases the quality of the products was higher than that of those processed by heat.

**Table 3:** HHP inactivation of spores in different foods.

Food product	Microorganism	HHP conditions	Inactivation results	Reference
Carrot juice	*B. licheniformis*	400–600 MPa for 0–40 min at 40–60 °C	241 to 465 MPa (D value range 23.3 to 31 °C)	Tola and Ramaswamy [33]
Cooked chicken	*C. botulinum*	600 MPa for 2 min at 20 °C	600 MPa for 2 min at 20 °C, 2 log reduction	Linton *et al.* [34]
Orange juice	*A. acidoterrestris*	200–600 MPa for 1–15 min at 45–65 °C	600 MPa, D55 °C = 7 min; 200 MPa, D65 °C = 5.0 min	Silva *et al.* [35]
Tomato sauce	*B. coagulans*; *A. acidoterrestris*	100–800 MPa for 10 min at 25, 40, 60 °C	700 MPa for 10 min at 60 °C, 2 log reduction	Vercammen *et al.* [36]
Tomato pulp	*B. coagulans*	300–600 MPa for 0–39 min at 50–60 °C	600 MPa for 15 at 60 °C 5.7 log reduction	Zimmermann *et al.* [31]
Orange Juice	*A. acidoterrestris*	200–600 MPa for 10 min at 20–60 °C	600 MPa for 10 min at 50 °C, 3 log reduction	Hartyáni *et al.* [37]
Milk	B.sporothermodurans	300–500 MPa for 10–30 min at 30–50 °C	495 MPa for 30 min at 49 °C, 5 log reduction	Aouadhi *et al.* [38]

At present, methods to germinate spores before HHP treatment are under study. Exist different methods for germination of spores such as combining extremely high pressure and temperature, methods that involves using low or medium pressure (150–300 MPa), temperature, and other factors as single amino acids, sugars, asparagine, glucose, fructose to germinate the spores and produce bacterial vegetative cells, after which the bacterial vegetative cells are inactivate using HHP [39,40]. In addition, there are other germinant agents, which include lysozyme, salts, and cationic surfactants such as dodecylamine, that can be used in combination with high pressure. It is important to point out however that the spores of proteolytic *Clostridium botulinum* and *Clostridium sporogenes* germinate in response to l-alanine but not to universal germinant AGFK (a mixture of l-asparagine, d-glucose, d-fructose, and potassium ions) or inosine [40,41,42,43]. This initial process can be followed by HHP treatment of 300–900 MPa at 30–60 °C [34,44].

According to the study carried out by Georget *et al.* [45] to germinate *Geobacillus stearothermophilus* spores under moderate high pressure in buffer *N*-(2-acetamido)-2-aminoethanesulfonic acid (ACES) applying a treatment of 200 MPa with temperature of 55 °C , an inactivation over 2 log10 was achieved after 5 min of treatment. A 200 MPa for 40 min at 55 °C treatment led an inactivation of 3 log reduction following the subsequent inactivation to 80 °C for 20 min. In case of the spores of *Clostridium botulinum* earlier studies in cooked chicken with 2% sodium lactate, showed that germination of spores occurred at 4 °C and a spore reduction in the initial inoculum of 1.7 log10 cfu/g with a treatment at 600 MPa for 2 min at 20 °C was achieved [38]. For the germination and inactivation of *Clostridium perfringens* spores in poultry meat, spores were incubated for 15 min at 55 °C with an addition of l-asparagine and potassium chloride, followed of a treatment of 568 MPa at 73 °C for 10 min achieving ~4 log reductions in the concentration of spores [33].

## 6. Effects of HHP on Proteins

HHP technology has been used fundamentally to reduce the microbial load and increase the safety and shelf life of treated foods with superior nutritional and sensory properties to those thermally treated. Nevertheless, the effect of HHP on proteins has raised interest and studies have been carried out to elucidate it. High Hydrostatic Pressure treatments affect the non-covalent links (ionic, hydrophobic, and hydrogen links) of proteins, which means that the secondary, tertiary, and quaternary structures can be unfolded and dissociated while the primary structure remains stable [46]. Messens *et al.* [47] reported that it is necessary to apply a pressure of around 150 MPa to observe changes in the quaternary structure, and it is necessary to apply more than 200 MPa to significantly modify the secondary and tertiary structures. Owing to these changes, Liu *et al.* [48] and Tabilo-Munizaga *et al.* [49] studied the application of this technology to develop industrial applications to confer unique characteristics to foods (gel formation, emulsions, foams, new flavors and textures) or to seek a fat replacement. The possibility of using these new products as fat replacements has encouraged in-depth studies of stabilizing and gelling agents, agents that are usually incorporated in foodstuffs to give stability, texture, and palatability [50,51]. It is important to note that the changes depend directly on the type of protein used (disulfide bridges, linked by hydrophobic interactions, isoelectric points) and the HHP treatment (pressure, time, and temperature) [52,53]. All these studies make HHP a promising technology for the revalorization of waste and agro-industrial by-products.

According to He *et al.* [54], when proteins isolated from peanuts were treated at pressures between 50 and 200 MPa for 5 min the isolates increased their water-holding capacity (WHC) and oil-binding capacity (OBC), producing changes of interest in relation to protein properties. Additionally, the effect of HHP on milk proteins and whey has been studied in depth under various treatment conditions. The results indicated various changes in protein structure. Casein micelles experienced significant changes at pressures between 150 and 400 MPa and at a temperature of 20 °C [55]. However, a greater denaturalization of proteins from whey β-lactoglobulin and α-lactalbumin was observed at pressures higher than 100 and 400 MPa, respectively [47,55,56].

The effect of pressure on vegetable proteins has also been studied. Protein isolates from peanuts (5% w/v of protein) produced gels at 100 MPa for 5 min at 25 °C, while isolates from soya protein (9% w/v of protein) produced gels at 600 MPa for 5 to 10 min and a temperature of 33.5 °C [57,58]. For gelification of the isolates it was necessary to add CaCl_2_ at a concentration of 0.015–0.020 mol L^–1^, in accordance with the work reported by Maltais *et al.* [58], who indicated that calcium concentrations are very important and determine the final characteristics of gels. At low calcium concentrations filamentous gels occurred, while at high concentrations disordered phase separation gels or aggregates appeared.

## 7. Effect of HHP on Enzymes

There are two important regions in an enzyme, one responsible for recognizing the substrate and the other responsible for catalyzing the reaction when joined to the substrate. Minimal conformational change in the structure may completely affect the enzyme functionality.

Enzymes can be divided into two groups according to the effect of treatment by high hydrostatic pressure. In the first group are enzymes that are activated with pressures of 100–500 MPa, an activation that occurs only in monomeric proteins [59,60]. The second group includes enzymes that are inactivated when exposed to pressures higher than 500 MPa in combination with relatively high temperatures [61,62,63]. The main studies conducted on the effect of HHP on enzymes are based on the enzymes that are most often present in foodstuffs and produce deterioration of it or unacceptable sensory changes (Table 4). Among them we can highlight the enzymes peroxidase (POD), pectin methylesterase (PME), lipoxygenase (LOX), and polyphenol oxidase (PPO), as shown in the study carried out by Ludikhuyze *et al.* [64]. In general, polyphenol oxidase (PPO) and peroxidase (POD) are inactivated by applying a pressure equal to or greater than 400 MPa in combination with temperatures between 20 and 90 °C. Under these conditions, enzyme activity can be reduced by up to 50%, although the percentages may vary depending on the intrinsic properties of processed foods. It should be noted that the predictive models used in thermal inactivation are often inadequate to describe inactivation by HHP treatment [65].

**Table 4:** HHP inactivation of enzymes in different foods.

Food product	Enzyme	HHP conditions	Inactivation achieved	Reference
Jam	Pectin methylesterase (PME); Peroxidase (POD)	550–700 MPa for 2.5–75 min at 45–75 °C	PME: 27%–40% POD: 51%–70%	Igual *et al.* [61]
Feijoa puree	Peroxidase (POD); Polyphenol oxidase (PPO); Pectin methylesterase (PME)	600 MPa for 5 min at 25 °C	POD: 78% PPO: 55.6% PME: 56%	Ortuño *et al.* [62]
Camarosa strawberry	Polyphenol oxidase (PPO)	600 MPa for 15 min at 34–62 °C	PPO: 82%	Sulaiman and Silva [65]
Fruit smoothies	Polyphenol oxidase (PPO)	600 MPa for 10 min at 20 °C	PPO: 83%	Keenan *et al.* [66]
Dry-cured ham	Glutathione peroxidase (GSHP*x*); Superoxide dismutase (SOD)	900 MPa for 5 min at 12 °C	GSHP*x*: 44.2% SOD: 17.6%	Clariana *et al.* [67]
Strawberry pulps	β-Glucosidase; Polyphenol oxidase (PPO); Peroxidase (POD)	400–600 MPa for 5–25 min at 25 °C	β-Glu: 41.4% PPO: 74.6% POD: 74.6%	Cao *et al.* [68]

## 8. Some Industrial Applications of HHP

HHP technology has become a commercially implemented technology in fruit juice processing, spreading from its origins in Japan to the USA and Europe, and now Australia, with worldwide utilization increasing almost exponentially since 2000. In the U.S., Genesis Juice Corp.^®^ processes eight types of organic juices by HHP, including apple, carrot, apple-ginger, apple-strawberry, ginger lemonade, strawberry lemonade, a herbal tea beverage, and apple- and banana-based smoothies, other company of high interest by its increment in sales in U.S., is Suja™, situated in San Diego, CA, produces a variety of mixture vegetable and fruits juices. European companies presently employing this technology in fruit juice processing include Invo^®^ making smoothies in Spain, UltiFruit^®^ making orange and grapefruit juices and a mixture of strawberry-orange juice in France, Frubaça^®^ manufacturing various fruit-based beverages in Portugal, Juicy Line-Fruity Line^®^ in Holland, Beskyd Frycovice, a.s^®^ manufacturing mixtures of broccoli-apple-lemon and broccoli-orange-lemon in the Czech Republic, ATA S.P.A.^®^ manufacturing carrot and apple juices in Italy, and Puro^®^ commercializing smoothies in the UK.

Regarding processing conditions, treatments are optimized at a pressure level of 600 MPa in combination with moderate heat. In addition, due to the special characteristics of fruit juices, (nutritional components, flavor) and the perception by the consumer as a healthy food, quantities ranging from 500 to 2000 kg/h can be produced to satisfy current consumer demand considering the current capacities of industrial equipment. Shelf lives are estimated at *ca.* 10–35 day under refrigeration conditions, depending on the type of juice. Products are sold in supermarket chains, specialty and gourmet stores, and food services providing fruit preparations and dressings. Two main packaging formats are used, a small volume containing 250 mL, corresponding to a single portion, and a larger format containing 1 L.

**Figure 1:**
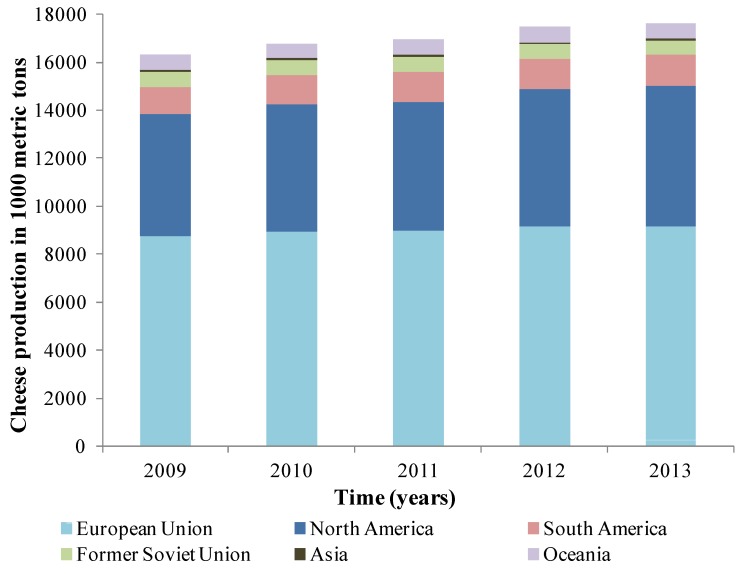
Cheese production for selected countries (in 1000 metric tons). Source: USDA [73].

One application of HHP that has great appeal is the stabilization of fresh cheese due its global consumption and the increase in global production of 3.5 million tons in the last 10 years (Figure 1), the cheese belongs to the ready-to-eat (RTE) food group, this product is characterized by special physical and chemical properties such as a near neutral pH, high water activity of 0.97, and high relative humidity, and is very prone to contamination from pathogens such as *Staphylococcus aureus*, *Listeria monocytogenes*, *Salmonella* spp., *Escherichia coli* O157:H7 [69,70], and spoilage microorganisms, such as molds and yeasts. Although currently this type of fresh cheese is made from pasteurized milk [71], the microbial recontamination occurs during subsequent processes, commonly in the stages of handling and packaging [72]. That is why high hydrostatic pressure technology could be of great interest in the microbiological stabilization of this product, avoiding high annual losses from foodborne diseases in which fresh cheeses are involved and rejections due to spoilage.

## 9. Conclusions

HHP treatment has proven to be an effective technology to reduce the microbial load of foods for both pathogenic and spoilage microorganisms with minimal impact on the initial quality of the foods. To apply HHP to food preservation, various parameters such as time, pressure, temperature, and pH should be considered because these parameters determine the optimum pressure intervals for microbial inactivation. Likewise, a combined treatment of moderate temperature and HHP has proven to have great potential both for the inactivation of microorganisms and enzymes and for the development of new products due to the modification of proteins of animal or vegetable origin.
